# Transcranial direct current stimulation leads to faster acquisition of motor skills, but effects are not maintained at retention

**DOI:** 10.1371/journal.pone.0269851

**Published:** 2022-09-13

**Authors:** Nirsan Kunaratnam, Tyler M. Saumer, Giovanna Kuan, Zacharie Holmes, Dana Swarbrick, Alex Kiss, George Mochizuki, Joyce L. Chen

**Affiliations:** 1 Rehabilitation Sciences Institute, University of Toronto, Toronto, Ontario, Canada; 2 Heart and Stroke Foundation Canadian Partnership for Stroke Recovery, Sunnybrook Research Institute, Toronto, Ontario, Canada; 3 Faculty of Kinesiology and Physical Education, University of Toronto, Toronto, Ontario, Canada; 4 Evaluative Clinical Sciences, Sunnybrook Research Institute, Toronto, Ontario, Canada; 5 School of Kinesiology and Health Science, York University, Toronto, Ontario, Canada; BG-Universitatsklinikum Bergmannsheil, Ruhr-Universitat Bochum, GERMANY

## Abstract

Practice is required to improve one’s shooting technique in basketball or to play a musical instrument well. Learning these motor skills may be further enhanced by transcranial direct current stimulation (tDCS). We aimed to investigate whether tDCS leads to faster attainment of a motor skill, and to confirm prior work showing it improves skill acquisition and retention performance. Fifty-two participants were tested; half received tDCS with the anode on primary motor cortex and cathode on the contralateral forehead while concurrently practicing a sequential visuomotor isometric pinch force task on Day 1, while the other half received sham tDCS during practice. On Day 2, retention of the skill was tested. Results from a Kaplan-Meier survival analysis showed that participants in the anodal group attained a pre-defined target level of skill faster than participants in the sham group (χ2 = 9.117, *p* = 0.003). Results from a nonparametric rank-based regression analysis showed that the rate of improvement was greater in the anodal versus sham group during skill acquisition (F(1,249) = 5.90, *p* = 0.016), but there was no main effect of group or time. There was no main effect of group or time, or group by time interaction when comparing performance at the end of acquisition to retention. These findings suggest anodal tDCS improves performance more quickly during skill acquisition but does not have additional benefits on motor learning after a period of rest.

## Introduction

Performance improves with practice and practice takes time [1]. Depending on the motor skill we want to learn, whether learning to knit or to play a piano concerto, it may take days, months and even years to become proficient. Transcranial direct current stimulation (tDCS) is a safe [2] and non-invasive approach used to electrically stimulate the cortex via electrodes placed on the scalp [3]. It can potentially enhance motor skill learning in neurotypical individuals [4,5] and in those with neurological disorders, such as stroke [6,7]. Evidence from a meta-analysis suggests that people who receive tDCS to motor cortex during motor skill practice have better performance than those who receive sham tDCS [8]. What is unclear is whether the pairing of motor skill practice with tDCS enables the learner to acquire skills faster than if sham tDCS was applied.

It is thought that tDCS enhances motor skill learning through the modulation of corticospinal excitability, which leads to the induction of long-term potentiation, a mechanism that underlies learning. A seminal study showed that tDCS to primary motor cortex (M1) modulates its corticospinal excitability, as indexed by the motor evoked potential, measured with transcranial magnetic stimulation [9]. The effects of the stimulation are polarity dependent–corticospinal excitability is relatively increased when the positively charged anode electrode is placed over M1 [9–11]. The current induces a relative depolarization of a neuron’s membrane potential, increasing excitatory post-synaptic potentials leading to the induction of long-term potentiation [12–18]. In contrast, corticospinal excitability is relatively decreased when the negatively charged cathode electrode is placed over M1 [9–11,19]. In this context, the current induces a relative hyperpolarization of a neuron’s membrane potential, decreasing excitatory post-synaptic potentials leading to the induction of long-term depression [14,18,20,21]. It is also important to note that effects of tDCS on corticospinal excitability may be non-linear [22]. Taken together, findings from meta-analyses support the notion that tDCS with the anode on M1 leads to a relative increase in corticospinal excitability, and vice versa when tDCS is applied with the cathode on M1 [23,24]. Narrative reviews of the literature also support the notion that tDCS and other forms of non-invasive brain stimulation can induce long-term potentiation and depression [14,25].

Thus, it is thought that when anodal tDCS is paired with practice, motor skill learning can be further enhanced [18,26,27]. Across a variety of motor tasks, anodal tDCS to M1 applied during task practice improves motor performance more than practice with sham tDCS, when evaluated in a single session [28–31] or across multiple sessions [32–35]. To the best of our knowledge, there is one meta-analysis that synthesizes the motor sequence learning literature [8]. Findings suggests that the effects of anodal tDCS to M1 on learning are variable; effect sizes ranged from 0.15 to 1.68, depending on the outcomes assessed (e.g. speed versus accuracy), whether single versus multiple sessions were applied, and whether effects were evaluated during skill acquisition versus retention [8]. Other meta-analyses synthesizing effects of anodal tDCS to M1 on motor task execution more generally (e.g. response times, time to finish task, etc.) in younger [23] and older [36] adults, yield effect sizes of 0.92 and 0.72, respectively.

Prior work showed that a greater proportion of chronic stroke survivors who received anodal tDCS required less amount of training on the sequential visuomotor isometric pinch force task (SVIPT), to reach a target skill level compared to people who received sham tDCS [37]. To the best of our knowledge, it is unknown whether these findings would be the same in participants who identify as neurotypical. Our research is motivated by prior work where 5 days of consecutive motor skill practice on the SVIPT paired with anodal tDCS to M1 led to greater performance improvements and skill retention, in comparison to effects of practice paired with sham tDCS [33]. Visual inspection of the data presented in the paper suggests that it may take approximately 3 additional days of practice for motor performance of the sham tDCS group to be similar to that attained by the anodal tDCS group by the end of day 1. However, this was not statistically evaluated since it was not part of their research aim [33].

Therefore, the main objective of our study is to determine whether practice with anodal tDCS to M1 leads to faster attainment of a target level of skill compared to practice with sham tDCS, in neurotypical individuals. We hypothesized that participants who receive anodal tDCS during skill acquisition of the SVIPT will require less amount of practice to attain a target level of skill compared to participants who receive sham tDCS. The secondary objective is to replicate findings that anodal tDCS to M1 leads to better performance compared to sham tDCS. We hypothesize that participants who receive anodal tDCS to M1 while practicing the SVIPT, will have better performance during skill acquisition and at 24-hour retention, compared to participants who receive sham tDCS.

## Materials and methods

### Participants

Fifty-five participants who identified as neurotypical between 18 to 44 years old were recruited from the general public through poster advertisements at Sunnybrook Health Sciences Centre (Sunnybrook) and The University of Toronto (UofT), and by word of mouth. Of the fifty-five participants, data from three participants were excluded. In one participant, we could not locate the M1 hotspot using transcranial magnetic stimulation (TMS). Data from two participants were withdrawn as it was subsequently determined that they were tested with an incorrect version of the motor task. A total of fifty-two participants completed the study, Sunnybrook (n = 26) and UofT (n = 26).

To determine eligibility, participants were first screened over the phone for inclusion and exclusion criteria. Participants were included in the study if they were right-handed, as assessed with the Edinburgh Handedness Inventory [38], and between 18 and 44 years of age. The upper limit of 44 years was determined based on prior work suggesting sequence learning differs in individuals after 44 years of age [39]. Participants were excluded if they had contraindications to receiving non-invasive brain stimulation. We used a consensus-based screening questionnaire [40] and added the following additional exclusion criteria: family history of epilepsy, convulsions or seizures; neurological or psychiatric disorder; prior surgery to the brain; metal in the body; prior participation in any non-invasive brain stimulation study. Participants provided written informed consent before any experimental procedures began. This research study was approved by the Sunnybrook Research Institute Ethics Board (#2122) and The University of Toronto Human Research Ethics Board (#37719). Participants were compensated for their time. The study was registered at clinicaltrials.gov NCT03249961.

### Study design

**Fig 1:**
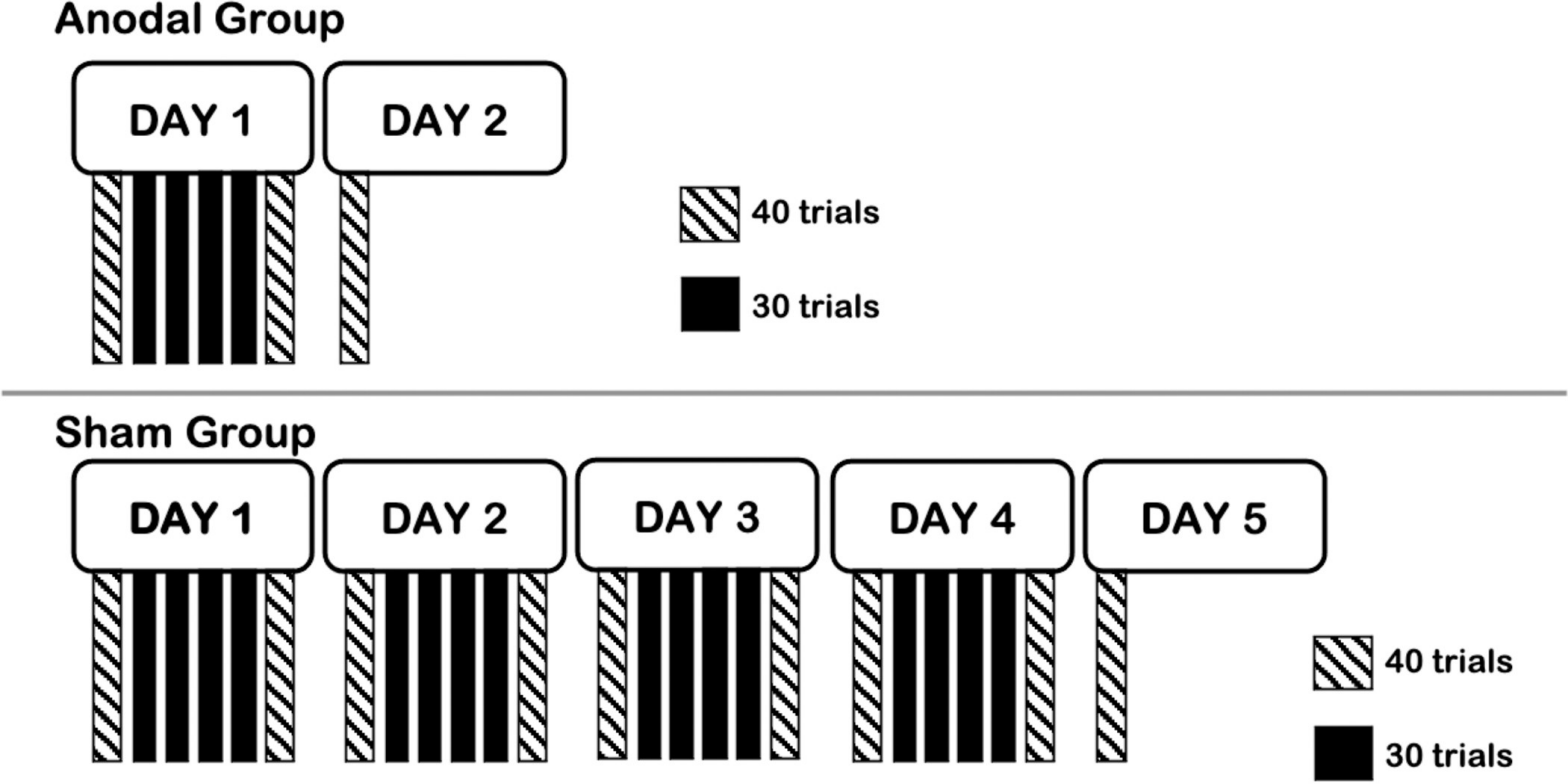
Study design. Study protocol for participants in the anodal and sham tDCS groups. Participants in the anodal tDCS group (Anodal Group) participated over 2 days, and participants in the sham tDCS group (Sham Group) participated over 5 days. In each day, participants performed the sequential visuomotor isometric pinch force task (SVIPT) with and without tDCS. Each black bar represents one block of practice that comprised 30 trials of practice on the SVIPT, with tDCS (anodal or sham) applied concurrently. Each hatched bar represents one block of practice that comprised 40 trials where no tDCS is applied.

Participants were randomized to receive anodal or sham tDCS via Research Randomizer software (https://www.randomizer.org/). Participants in the anodal tDCS group attended 2 sessions approximately 24 hours apart (Fig 1). In the first session, day 1, ‘skill acquisition’, participants performed the SVIPT (described in section 2.4) while concurrently receiving anodal tDCS (described in section 2.5). This allowed us to assess effects of online tDCS on the practice of a motor task. In the second session, day 2, “skill retention”, participants performed the SVIPT without tDCS. This allowed us to assess the relative permanency of performance [41]; are the effects of practice with tDCS from the first session, maintained the next day?

Participants in the sham tDCS group attended 5 sessions, each approximately 24 hours apart (Fig 1). In each of the first 4 sessions, days 1 to 4, participants performed the SVIPT while concurrently receiving sham tDCS. In the fifth session, day 5, participants performed the SVIPT without tDCS. Thus, the sham tDCS group is engaged in more sessions than the anodal tDCS group. This design allowed us to address the first study objective of whether the anodal tDCS group attains a target level of skill performance more quickly than the sham tDCS group. In another words, how many more blocks or sessions of practice were required for the sham tDCS group to reach the same target level of performance. To address the second study objective, we compared performance on days 1 and 2 between the two tDCS groups. This allowed us to assess whether there were any differences in skill acquisition and retention.

#### Task block and trial information

Participants performed 3 warm-up trials of the SVIPT that were not included in the analyses. The number of blocks of practice and number of trials per block is based on prior work [33]. On day 1 for the anodal tDCS group, participants completed 200 trials of the SVIPT, divided into 6 blocks. The first and last blocks (blocks 1 and 6) entailed 40 trials each, and the middle blocks (blocks 2, 3, 4, and 5) entailed 30 trials each (Fig 1). A rest period of 1 minute was provided between blocks. Anodal tDCS was applied only during the middle blocks, based on prior work [33]. On day 2, participants in the anodal tDCS group completed one block of 40 trials; no tDCS was applied. This allowed us to assess retention effects by comparing blocks with no tDCS (Day 1 block 6 versus Day 2 block 1).

Participants in the sham tDCS group underwent an identical protocol as participants in the anodal tDCS group with the following exceptions. First, sham tDCS was applied. Second, on each of days 1 through 4, 200 trials of the SVIPT were performed with the same block and trial design described above for the anodal tDCS group. On day 5, participants completed one block of 40 trials with no tDCS.

#### Blinding

This is a single blind study as only participants were blind to the study design including tDCS parameters. The consent form indicated participants would be randomly allocated to one of two groups, however, participants were not told whether they would receive anodal or sham tDCS. They were informed that researchers would be testing different types of stimulation and different amounts of practice. It was not possible to blind the experimenter since the sham tDCS group participated in more sessions than the anodal tDCS group.

### Questionnaires

We administered questionnaires before and after each session to characterize the study population, to understand how different factors may affect performance, and to understand reasons for potential outliers. We assessed: sleep duration of previous night, sleep quality, alertness, pain, overall fatigue, muscle soreness, consumption of coffee, and amount of physical activity. Participants were instructed to not consume coffee or exercise just prior to each study session. However, some participants had sessions later in the day and were not able to avoid their caffeine intake or exercise regimen. Before and after each training session, we also administered the Positive and Negative Affect Scale (PANAS) [42] to evaluate tDCS effects on mood [33,43]. In addition, a tDCS debriefing questionnaire was administered after each training session. Participants were asked if they experienced mild adverse events associated with tDCS [2]: itching, tingling, pinching, pain, burning, warmth, fatigue, headache, dizziness, discomfort. If so, they qualified the experience according to the following scale: none, mild, moderate, considerable, or strong. We also asked whether these mild adverse events affected their performance, and what tDCS condition they thought they received.

### Sequential visuomotor isometric pinch force task (SVIPT)

**Fig 2:**
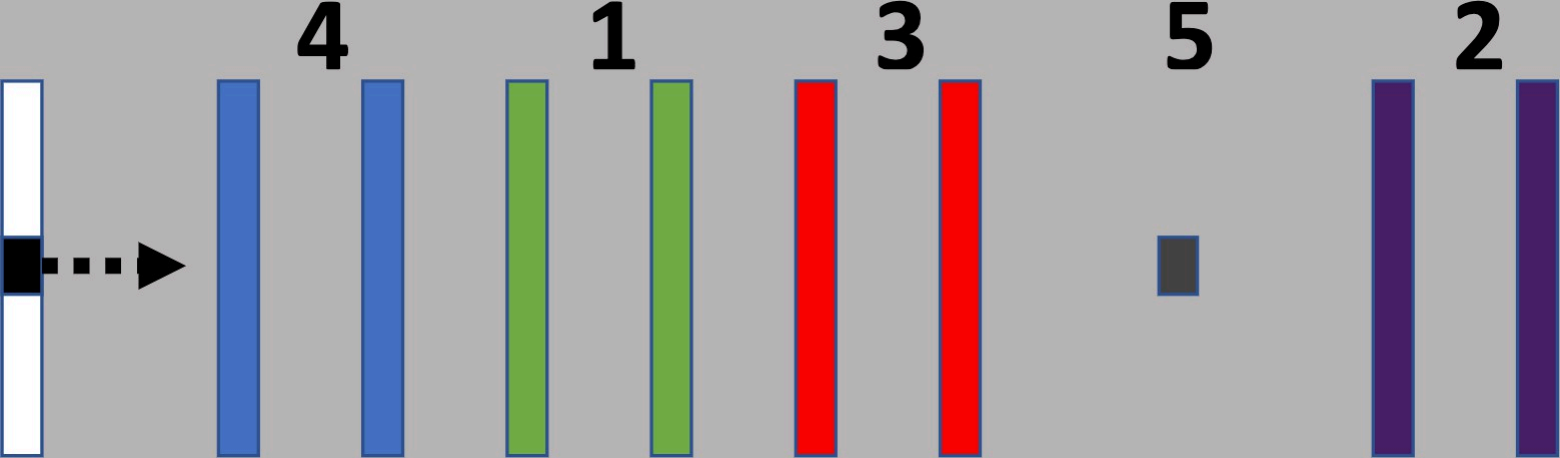
Sequential visuomotor isometric pinch force task. Participants navigate the on-screen cursor between the Home target (black square on the left of the screen) and four other targets, in a sequential order, ending at Target 5.

Participants performed the SVIPT, following previously reported methods [33]. This task has been used in prior work and thus enables us to compare our findings [43–45]. Participants were seated in an armless chair approximately 50 cm in front of a 15.6 inch Acer laptop monitor. The SVIPT was displayed using LabView 2013. Participants held a force transducer (Dacell, Model UU3 5kg force load cell) between the first digit (thumb) and lateral aspect of the middle phalanx of the second digit (index finger). Participants pinched the force transducer with their index finger and thumb to control an on-screen cursor that navigated between a Home target and 5 other targets numbered 4-1-3-5-2, left to right (Fig 2). Participants were required to hit the targets in the following order: Home-1-Home-2-Home-3-Home-4-Home-5. The order of targets presented remained the same across all sessions.

Targets 1 through 4 have two bars each, referred to as a “gate.” The cursor was required to be in-between each target’s gate to count as correct. Target 5 was the last target in a trial and therefore had one bar. To end the trial, participants maintained the cursor over Target 5 for 150 ms. A “GO” cue appeared at the beginning of each trial to indicate the start of the trial. A “STOP” cue appeared after the participant reached Target 5. There was a delay of 2,000 ms between trials. Trial duration was set to 30,000 ms. If the trial was not completed in this time, the trial ended, and the next trial began. Participants were instructed and reminded to not squeeze the force transducer prior to the “GO” cue. If this occurred and resulted in the cursor position being at or greater than Target 1 at cue onset, these trials were considered “invalid trials” and removed from analysis.

The amount of force applied to the transducer was normalized across all participants to ensure each participant applied the same relative pinch force during task performance for all sessions. Participants performed a maximum voluntary contraction (MVC), pinching the force transducer with as much force as possible, for three times. Following previously reported procedures [33], we took 40% of the highest MVC value to represent the amount of force needed to move the cursor to the farthest rightward target (i.e. Target 2). The amount of force required to reach the other targets was then logarithmically scaled to increase task difficulty.

### Transcranial direct current stimulation

#### Localizing primary motor cortex

Participants from Sunnybrook received TMS at the first session to determine the M1 hotspot from where we placed the anode electrode for tDCS, and to determine the resting motor threshold. TMS pulses were delivered using a D702 figure of eight shaped coil (The Magstim Company Limited, Whitland, UK), connected to a Magstim 2002 monophasic stimulating unit. The coil was held at an angle approximately 45% to the midsagittal line, positioned in a posterior to anterior orientation. The electromyography (EMG) signals were sampled at 1000 Hz by an analog-to-digital interface (Power 1401 mkII, Cambridge Electronic Design, Cambridge, UK). The amplification of electrical signal was set to 5000x, and band-pass filtered at 10 Hz– 1000 Hz online using an amplifier (Model QP511, Grass Technologies, West Warwick, RI, USA). Muscle activity was collected using data acquisition software (Spike2 Version 7.17, Cambridge Electronic Design, Cambridge, UK).

We placed the TMS coil over the C3 region of the scalp identified with the international 10–20 electroencephalography system [46]. The experimenter used the C3 region as a starting point and moved the coil systematically a few centimeters in each direction to locate the M1 hotspot. The area that consistently elicited the greatest motor evoked potential and an observable “twitch” in the right first interosseous muscle was determined to be the M1 hotspot. TMS was also used to determine the resting motor threshold at the M1 hotspot. The resting motor threshold is an indicator of cortical excitability, and is the lowest stimulus intensity on the TMS stimulator output required to elicit a motor evoked potential response at rest of ≥ 50μV microvolts in 5 out of 10 consecutive trials [47].

Participants enrolled from UofT were recruited at a later timepoint and it was not feasible for them to undergo TMS at the UofT site. Therefore, tDCS electrode placement was determined for UofT participants using the international 10–20 system, which is commonly done when TMS is not available [48]. As noted previously, one participant from Sunnybrook was excluded because the TMS hotspot could not be located. Retrospectively the 10–20 system could have been used on this participant. However, at that time of testing, we had not anticipated involving the UofT site and thus wanted to maintain consistency in the methodology.

#### tDCS parameters

We used the DC-Stimulator Plus device and accessories (electrodes, sponges, wires, headstrap) manufactured by NeuroConn GmbH (Ilmenau, Germany). This product is commonly used in research studies that apply tDCS [11,49,50]. It is battery driven with a maximum output of 4.5 mA. Two electrodes, each 5x5 cm^2^ in size were placed inside similar size sponges. The sponges were saturated with saline solution to increase conductance of electrical currents and reduce any side-effects (e.g., tingling, burning sensations, etc.). To minimize any inter-individual variability from different amounts of saline solution applied to the sponges, all participants received 12 mL of saline solution to each sponge. tDCS was applied through two wired electrodes placed on the surface of the scalp. The center of the anode electrode was placed over the M1 hotspot (Sunnybrook participants), or C3 area (UofT participants). The cathode electrode was placed on the contralateral supraorbital area.

For the anodal tDCS group, participants received 1 mA tDCS for 20 minutes. The tDCS parameters (including electrode size) were chosen for the following two reasons. First, we aimed to replicate findings by Reis et al [33] which motivated the secondary aim of our study. Second, these parameters have been commonly applied in prior work [8,26], which allows us to compare our findings to this literature. The current was ramped up over 30 seconds at the beginning and ramped down over 30 seconds at the end of the 20 minute window. Participants in the sham tDCS group received current that ramped up to 1 mA over 30 seconds, stayed on for 40 seconds, and ramped down over 30 seconds. This allowed participants in the sham tDCS group to experience similar sensations as the anodal tDCS group. The electrode montage remained on the head for the remaining 20 minutes with continuous monitoring of impedance.

### Dependent measures

The dependent measures were analyzed in Python using a custom script which is openly available on Github: https://github.com/dana-and-monsters/svipt-task-analysis/blob/main/SVIPT-Analysis.py.

#### Movement time

Movement time is defined as the time between a participant’s response to the Go cue, defined as the time the cursor reached 5% of its peak velocity, to the completion of a trial where the cursor stops at Target 5. We used this approach to ensure we were capturing a participant’s goal-directed movement towards the first target, and not noise derived from a participant simply holding the transducer. Movement time was averaged across trials, for each block.

#### Error rate

Error rate is defined as the ratio between the number of errors to the total number of possible hits. Based on the scoring approach of prior work [33], any missed target in a single trial, resulted in that trial labelled as an error with a score of zero points. A missed target occurs when a participant over- or under-shoots the gates of that target. In contrast, a hit occurs when a participant navigates the cursor accurately between all four target gates within a trial. Error rate was averaged across trials, for each block.

#### Skill

Skill was our primary outcome measure, which incorporates both movement time and error rate. We applied the formula below previously derived [33] and set the b-value to b = 0.05424 to

$$\mathrm{Skill}=\frac{1-\mathrm{error}\;\mathrm{rate}}{\mathrm{error}\;\mathrm{rate}\;(\ln{\left(\mathrm{movement}\;\mathrm{time}\right)}^{b}}$$

allow for positive values of the Skill measure. Skill was calculated for each block of trials across all sessions.

### Statistical analyses

All statistical tests were run using IBM SPSS Statistics 24 (IBM, Armonk, USA) or SAS version 9.4 (SAS Institute, Cary, NC, USA). First, we evaluated the data for normality using the Shapiro-Wilk test, homogeneity and outliers. Non-parametric tests were performed if these assumptions were violated. Data points that were outside 3 interquartile range were defined as outliers and excluded from analyses [51]. For all multivariable models, multicollinearity among the predictor variables (defined as a tolerance statistic <0.4) was assessed prior to modeling. Should multicollinearity be found to be a concern, only one member of a correlated set of variables would be retained for the model.

### Demographic data

Age, sex, and handedness were assessed between anodal and sham tDCS groups using separate logistic regression adjusting for site.

### Baseline performance

Skill performance at Block 1 of Day 1 was compared between anodal and sham tDCS groups with an independent samples t-test (2-tailed) to determine whether the groups differed at baseline.

### Primary analysis: Does training with anodal tDCS leader to a faster attainment of skill?

We performed a Kaplan-Meier survival analysis and associated log rank test to compare groups. The survival analysis yields information on the number of participants in both groups who achieve a pre-determined “event”. The “event” is defined as the average Skill performance attained by the anodal tDCS group on Day 1 Block 6. For each participant in both groups, we determined the time-to-event, which is the number of blocks needed for that participant to reach the “event”. The outcome of interest from the survival analysis is median time-to-event, which occurs when half the participants reach the time-to-event.

### Secondary analyses: Does training with anodal tDCS lead to better performance?

We evaluated Skill acquisition on Day 1 using a nonparametric regression analysis based on the ranks of the observed data points [52]. The model included time (Blocks 1–6), group (anodal, sham tDCS) and a group by time interaction term as the key factors. The model also adjusted for clustering within sites as well as covariates such as age and exercise.

We evaluated Skill retention at 24 hours using a nonparametric regression analysis based on the ranks of the observed data points. The model included time (Block 6 on Day 1, Block 1 on Day 2), group (anodal, sham tDCS) and a group by time interaction term as the key factors. The model also adjusted for clustering within sites as well as covariates such as age and exercise. Although the sham tDCS group continued to practice over additional days, we assessed skill retention on Block 1 Day 1 (i.e. 24 hours after skill acquisition). This allowed us to compare skill retention between groups without the confound of the additional practice sessions that the sham group received. We also quantified the number of participants who increased, maintained, or decreased their performance from the last block on Day 1 to the first block on Day 2. Values of Skill on the first block of Day 2 that were greater than 10% of performance on the last block of Day 1 were categorized as performance increases, values lower than 10% were categorized as performance decreases, and values within 10% were qualified as no change or maintenance of performance.

## Results

Fifty-two neurotypical right-handed participants completed the study (Sunnybrook: n = 13 anodal tDCS, n = 13 sham tDCS; UofT: n = 13 anodal tDCS, n = 13 sham tDCS). According to the Shapiro-Wilk Test, normality was violated (*p*<0.005). Levene’s test revealed that variance of our primary dependent measure, Skill, was not significantly different (*p* = 0.264). We examined data in each block per group and removed outlier data points if they were outside 3 interquartile range. In the anodal tDCS group, a total of 6 datapoints were removed and in the sham tDCS group, a total of 28 datapoints were removed. There were more datapoints in the sham versus the anodal tDCS group because the former had more training sessions.

Invalid trials where the cursor position was at or greater than Target 1 at cue onset were also removed. In the anodal tDCS group, 21 out of 6,240 trials (0.34%) were invalid. In the sham tDCS group, 140 out of 21,840 trials (0.64%) were invalid.

### Demographic data

Demographic data are presented in Table 1. There was a significant difference in age: participants in the sham tDCS group were older than the participants in the anodal tDCS group across both sites (*p*<0.001). There was a significant difference in the number of participants that exercised prior to start of Day 1: more participants in the anodal tDCS group exercised prior to the start of Day 1 across both sites (*p*<0.001). Therefore, all the primary and secondary analyses were adjusted to include age and number of participants that exercised prior to the start of Day 1 as covariates. There was no significant difference in sex (*p* = 0.160) caffeine (*p* = 0.152), and handedness (*p* = 0.953) between the two groups and across sites. An independent samples t-test revealed no significant difference in the resting motor threshold between the anodal versus sham tDCS groups (*p* = 0.892).

**Table 1:** Demographic information.

	Sunnybrook Anodal (n = 13)	Sunnybrook Sham (n = 13)	UofT Anodal (n = 13)	UofT Sham (n = 13)	z or t-statistic, *p*-value
Age (years); Mean (SD)	24.23 (4.73)	26.31 (4.31)	22.54 (3.20)	27.31 (7.69)	z = 7.83, *p*<0.001
Sex (number of females); percentage in respective group	7 (54%)	7 (54%)	5 (38%)	6 (46%)	z = 1.40, *p* = 0.160
Number of participants that consumed caffeine on the same day prior to start of study; percentage in respective group	4 (31%)	8 (62%)	5 (38%)	5 (38%)	z = 1.43, *p* = 0.152
Number of participants that exercised prior to start of Day 1; percentage in respective group	2 (15%)	0 (0%)	6 (46%)	4 (31%)	z = 12.46, *p*<0.001
* Handedness (percentage); Mean (SD)	91.96 (9.43)	86.76 (16.53)	87.53 (13.76)	93.63 (11.30)	z = 0.11, *p* = 0.953
Resting motor threshold (percentage of maximum stimulator output); Mean (SD)	40.00 (7.27)	39.62 (7.03)	N/A	N/A	t = 0.137, *p* = 0.892

All analyses reported in this table were performed with logistic regression adjusting for site, except for resting motor threshold, for which we performed an independent samples t-test. SD: Standard deviation. *Laterality quotient assessed by the Edinburgh Handedness Inventory.

### Baseline performance

**Fig 3:**
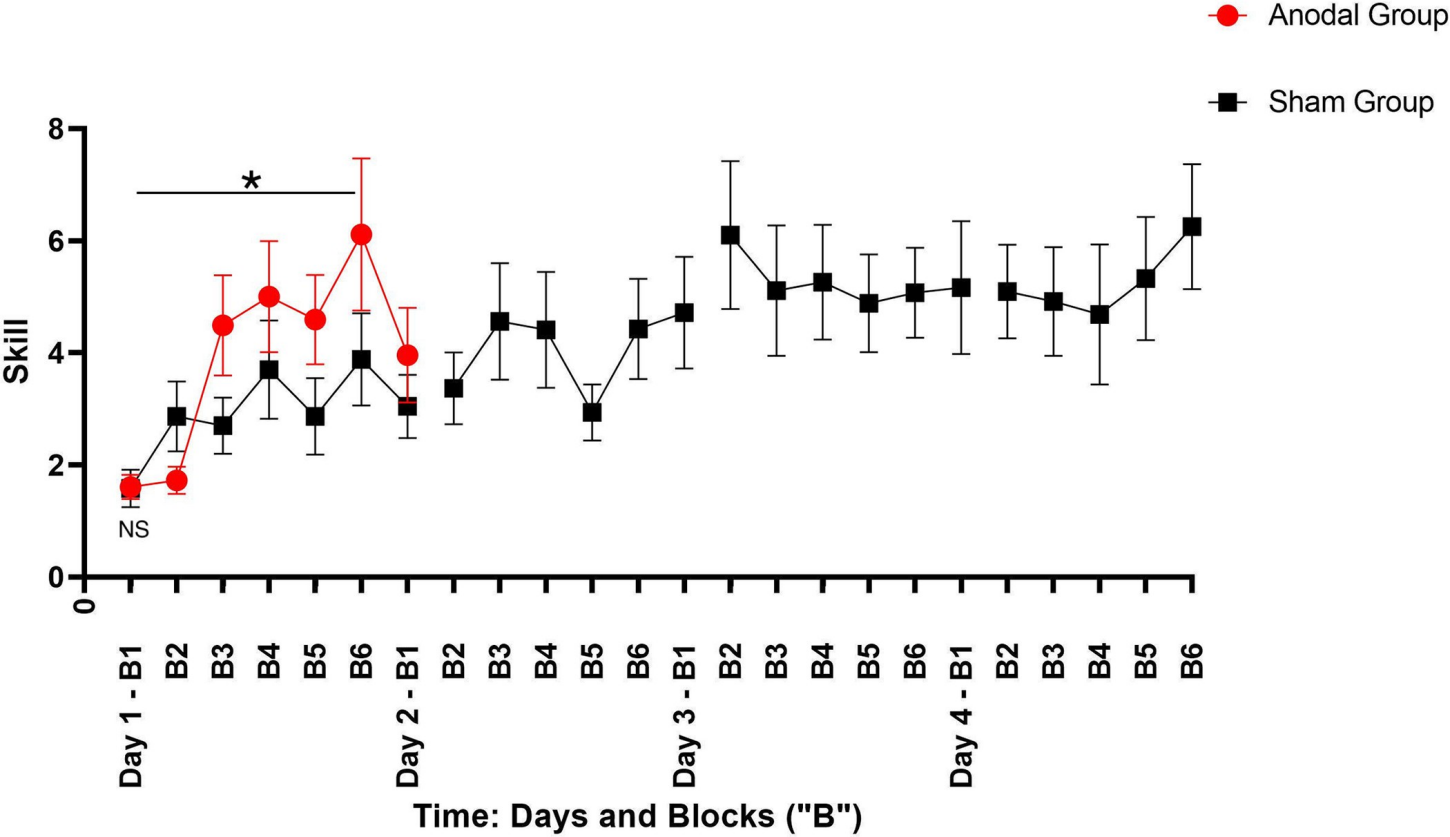
Performance across days of practice. Mean skill performance (y-axis) for each block of practice across days (x-axis). Bars represent standard error. Red circles represent data associated with performance of the anodal tDCS group (Anodal Group), and black squares represent data associated with performance of the sham tDCS group (Sham Group). On day 1 block 1, there is no significant difference (NS) in performance between the Anodal and Sham Groups (t = 0.234, *p* = 0.816). On day 1 across both groups, there is a significant improvement in performance across blocks of practice (F(1,249) = 49.21, *p*<0.001). The Anodal Group also had a significantly greater increase in performance across blocks of practice as compared to the Sham Group (F(1,249) = 5.90, *p* = 0.015), indicated by the star (*).

There was no difference in baseline Skill performance in both groups on Day 1 Block 1 (t = 0.234, *p* = 0.816) (Fig 3).

### Primary analysis: Does training with anodal tDCS lead to a faster attainment of skill?

**Fig 4:**
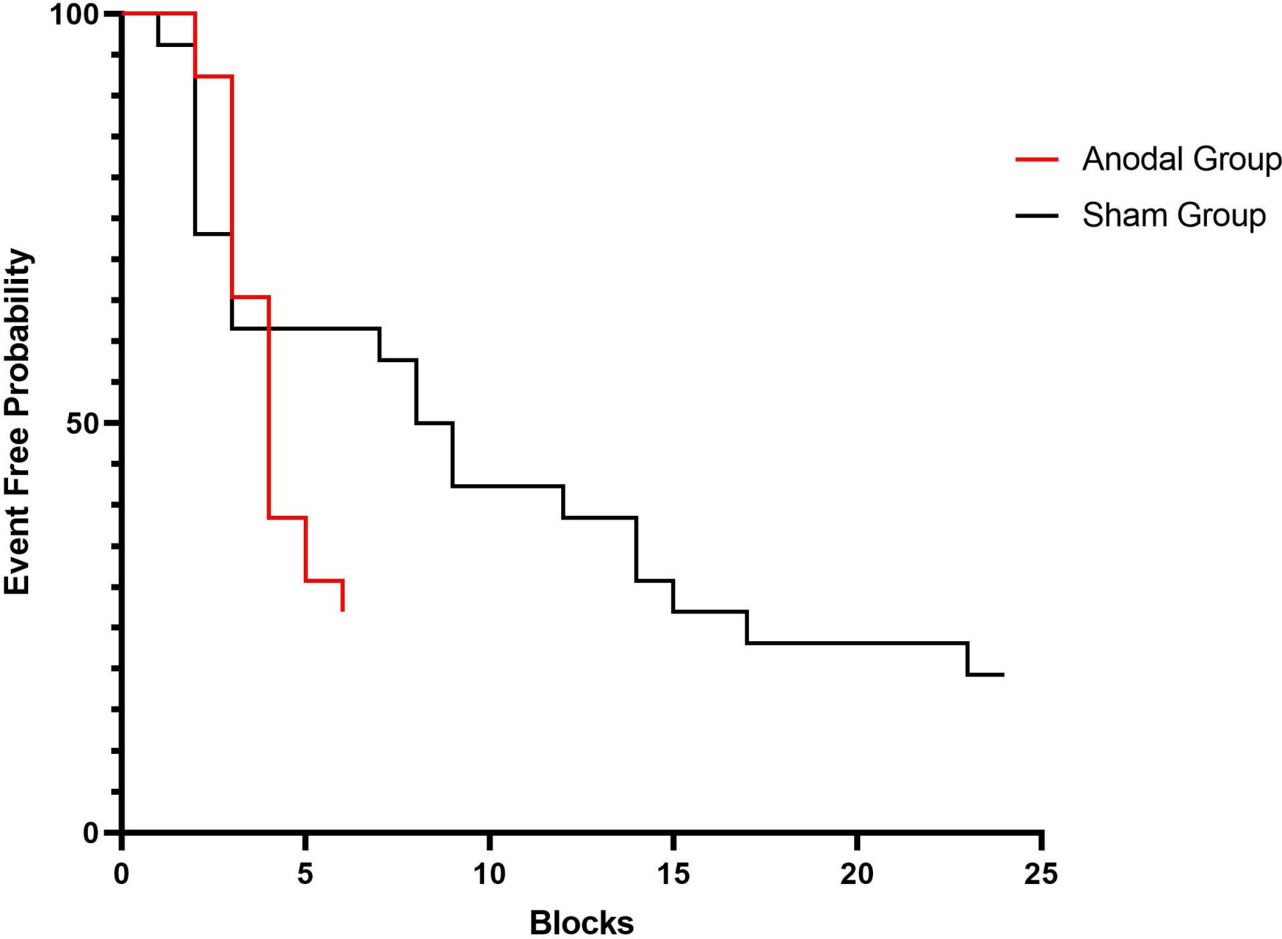
Learning probability. The y-axis corresponds to the percentage of participants who were unable to complete the event. The x-axis corresponds to the number of blocks of practice. At the start of the study, 100% of participants did not reach the event (i.e. y-axis “Event free probability” = 100%); this percentage drops as the study progresses over time. Fifty percent (i.e. y-axis “Event free probability” = 50%) of the anodal group (red line) completed the event by 4 blocks (i.e. x-axis “Blocks” = 4), whereas for the sham group (black line), 50% of participants completed the event by 8 blocks. These two curves are significantly different from each other (χ2 = 9.117, *p* = 0.003).

The event, which was defined as the average “Skill” performance on Day 1 Block 6 for the anodal tDCS group, was 4.02. There was a significant difference between the learning probability curves for the anodal and sham tDCS groups (χ2 = 9.117, *p* = 0.003, Fig 4). Thus, the anodal tDCS group achieved the event faster than the sham tDCS group. The median time-to-event for the anodal tDCS group was 4 Blocks; 16 out of 26 participants reached the event by Block 4. The median time to event for the sham tDCS group was 8 Blocks; 13 out of 26 participants reached the event by Block 8 (the equivalent of Day 2 Block 2). By the end of the first training session (Day 1 Block 6), 7 out of 26 participants in the anodal tDCS group did not reach the event, and 16 out of 26 participants in the sham tDCS group did not reach the event.

### Secondary analyses: Does training with anodal tDCS lead to better performance?

For skill acquisition, a nonparametric rank-based regression analysis revealed a significant time by group interaction where the anodal tDCS group had a greater increase of Skill over time (slope) than the sham tDCS group (F(1,249) = 5.90, *p* = 0.015) (Fig 3, see S1 Fig for individual performance curves). There was a main effect of time whereby both groups improved their Skill performance across blocks in Day 1 (F(1,249) = 49.21, *p*<0.001). There was no main effect of group (F(1,48) = 0.00, *p* = 0.995).

For Skill retention, a nonparametric rank-based regression analysis revealed no significant time by group interaction (F(1,48) = 0.41, *p* = 0.526) (Fig 3). There was no main effect of time (F(1,48) = 2.00, *p* = 0.164) between the last block on Day 1 and the first block on Day 2 (24-hour retention). In addition, there was no main effect of group (F(1,48) = 1.41, *p* = 0.238). In comparing performance across Days 1 and 2, 19 participants increased (n = 9 anodal, n = 10 sham), 24 decreased (n = 10 anodal, n = 14 sham), and 9 maintained (n = 7 anodal, n = 2 sham) their performance.

### Outcome of blinding and other questionnaires

Fifteen out of 26 participants in the anodal tDCS group correctly believed they received anodal tDCS after the session on Day 1; 11 incorrectly guessed they received sham tDCS. Nine out of 26 participants in the sham tDCS group correctly believed they received sham tDCS after the session on Day 1; 17 incorrectly guessed they received anodal tDCS. Mild adverse events are defined as mild symptoms for which no medical treatment is necessary [2]. Mild adverse events experienced after the session on Day 1 are reported in S1 Table. Qualitatively, both anodal and sham tDCS groups experienced none to mild adverse events. Factors that could affect performance on Day 1 are reported in S2 Table; qualitatively both groups reported similar states. Lastly, self-reported affect on Day 1 as measured by the Positive and Negative Affect scale are reported in S3 Table; qualitatively both groups report similar levels of affect.

## Discussion

The primary objective of our study was to determine whether practice with anodal tDCS to M1 leads to faster attainment of a target level of skill compared to practice with sham tDCS. We found that individuals who practiced the SVIPT with anodal tDCS to M1 on average, attained the target level of skill faster than those who practiced with sham tDCS. The secondary objective of our study was to compare the effect of anodal versus sham tDCS to M1 on skill acquisition and retention performance. We found the rate of performance improvements was greater over blocks of skill acquisition, in participants who received anodal versus sham tDCS. However, performance at retention was similar between groups. Together, our findings support the notion that anodal tDCS to M1 has some effect on motor skill performance but the effect does not persist over time.

Anodal tDCS to M1 during skill acquisition may reduce the amount of practice required to attain a pre-determined skill level. Our findings are similar to those previously reported where people with chronic stroke also required less practice to achieve a target skill level on the same SVIPT task when their practice was paired with anodal as compared to sham tDCS [53]. These findings could be of potential relevance to people wanting to make performance gains over a quicker period of time. For example, patients in rehabilitation hospitals could be discharged sooner, and athletes or musicians could accelerate their practice by achieving milestones in performance more quickly. Future work is needed to evaluate whether tDCS can accelerate performance on these naturalistic/real-world motor skills. It is also important to keep in mind that not everyone in the anodal group reached the target skill level by the end of training on Day 1. Hence, there was some variability in participant response. Variability in response to tDCS has been widely documented [54,55], and can be attributed to factors such as the state of the participant, as well as parameters of the tDCS protocol including intensity and duration of stimulation. We applied a commonly used tDCS protocol (1 mA, 20 minutes, 25 cm^2^ sponge size) also applied in prior research with the SVIPT task [8]. Protocols using the same or similar parameters yield variability in the neurophysiological (i.e. corticospinal) response as measured with the motor evoked potential [56–58], which could lead to variable behaviour.

We found that the rate of improvement during skill acquisition was greater for the anodal than sham tDCS group. However, there was no difference in performance at the retention test 24 hours later. Our findings replicate prior research that generally shows anodal tDCS to M1 improves motor performance more than sham tDCS [8,26]. When considering studies that employ the SVIPT task with anodal tDCS, the findings are inconsistent across studies. A seminal study by Reis found anodal tDCS to M1 enhanced offline gains across days of skill acquisition but not online (i.e. skill acquisition) performance, relative to sham tDCS [33]. In contrast, later work showed improvements in performance during online skill acquisition [45,53,59] but not offline gains [8,45,53], which is in line with what we found. Note that we only tested the effects of tDCS after one session of practice (i.e. Day 1), and effects may be more evident when tDCS is paired with skill acquisition over multiple days [8]. Variability in performance at retention relative to end of acquisition was also evident; almost half of the participants decreased their performance, and the other half of the participants increased or maintained their performance. It is not clear what could account for this variability. Qualitatively, both anodal and sham tDCS groups reported similar levels of affect, state, and mild adverse events experienced, and did not appear aware of which tDCS condition they received.

Together our findings show that anodal tDCS to M1 may hasten and improve the effects of practice, but these effects may not persist for everyone at skill retention. The topic of variability in response to tDCS has been much discussed and is still unresolved [5,54]. The parameter space is large and many factors such as sex, age, neuroanatomy, etc. affect how any individual responds [54]. The brain also has homeostatic plasticity mechanisms that regulate the induction of long-term potentiation (LTP)-like plasticity [25,60]. Thus, we speculate that there may be limits as to how much an external stimulus (i.e. tDCS) could have on facilitating cortical plasticity. Motor learning leads to the induction of LTP-like plasticity, which subsequently limits or occludes further LTP-like plasticity from taking place via another training bout, or plasticity-inducing brain stimulation protocol [61]. There are individual differences in the degree to which learning occludes the ability of synapses to become further potentiated [62]. This could partly explain why tDCS has on average, an immediate, online effect on performance, but that these effects do not persist for everyone. Continued research is required to test whether there are limits to how much we can artificially/externally induce neuroplasticity.

There are a few limitations of our work. First, we could not use TMS for participants tested at the UofT site, so targeting of M1 may not have been precise. Second, we paired tDCS with the SVIPT task, which is widely used in studies of motor learning [8]. Recent evidence suggests that the cerebellum is also involved in this task and thus the sole targeting of M1 may not be as optimal [63]. Third, we did not screen for whether participants had any musculoskeletal injuries in their upper extremity, which could have affected performance. However, experimenters observed that participants did not present with any overt symptoms related to their upper extremity. Further, reported perceptions of pain, fatigue and muscle soreness did not suggest that these factors could have influenced performance (See S2 Table). Fourth, the experimenters were not blinded to the study design and thus knew which participants received sham versus tDCS with the anode on M1. It was impossible to blind the experimenters since participants who received tDCS with the anode on M1 were only required to attend two days of testing. In contrast, participants who received sham tDCS attended five days of testing.

## Supporting information

S1 Fig Individual performance curves. Mean skill performance (y-axis) for each block of practice across days (x-axis). Each line represents one participant’s mean skill performance. Red represent data for the anodal transcranial direct current stimulation group; black represent data for the sham transcranial direct current stimulation group.
(TIF)

S1 Table Reported mild adverse events of transcranial direct current stimulation. Mild adverse events reported at debriefing after the session on Day 1 for both anodal and sham transcranial direct current stimulation (tDCS) groups. Participants were also asked if they thought their performance was affected by the tDCS. Participants scored according to the following scale: 1 = none; 2 = mild; 3 = moderate; 4 = considerable; 5 = strong. Data represent mean (standard deviation).
(DOCX)

S2 Table Factors that may affect performance. Mean (SD) ratings for factors that could affect performance, measured before (Pre) and after (Post) the session on Day 1 for both anodal and sham transcranial direct current stimulation (tDCS) groups. Sleep quality was rated on the following scale: 1 = poor; 2 = adequate; 3 = normal; 4 = good; 5 = excellent. The visual analogue scale (VAS) on a range from 1 to 7 was used to assess other factors. Alertness was rated as 1 = low alertness to 7 = high alertness; pain was rated as 1 = no pain to 7 = severe pain; fatigue was rated as 1 = not tired to 7 = extremely tired; muscle soreness was rated as 1 = no muscle soreness to 7 = muscles extremely sore.
(DOCX)

S3 Table Self-reported measures of affect. The Positive and Negative Affect Schedule (PANAS) to screen for mood was administered before (Pre) and after (Post) the session on Day 1 for both anodal and sham transcranial direct current stimulation (tDCS) groups. Data represent mean (SD). A higher “positive” score indicates a more positive affect; a lower “negative” score indicates less of a negative affect. Scores range from 10–50.
(DOCX)

S1 Raw data (XLSX)
