# Supplementary figures and images for: Transcranial direct current stimulation leads to faster acquisition of motor skills, but effects are not maintained at retention
Source: PLoS One. 2022 Sep 13;17(9):e0269851. doi: 10.1371/journal.pone.0269851 (PMC9469971; doi:10.1371/journal.pone.0269851)

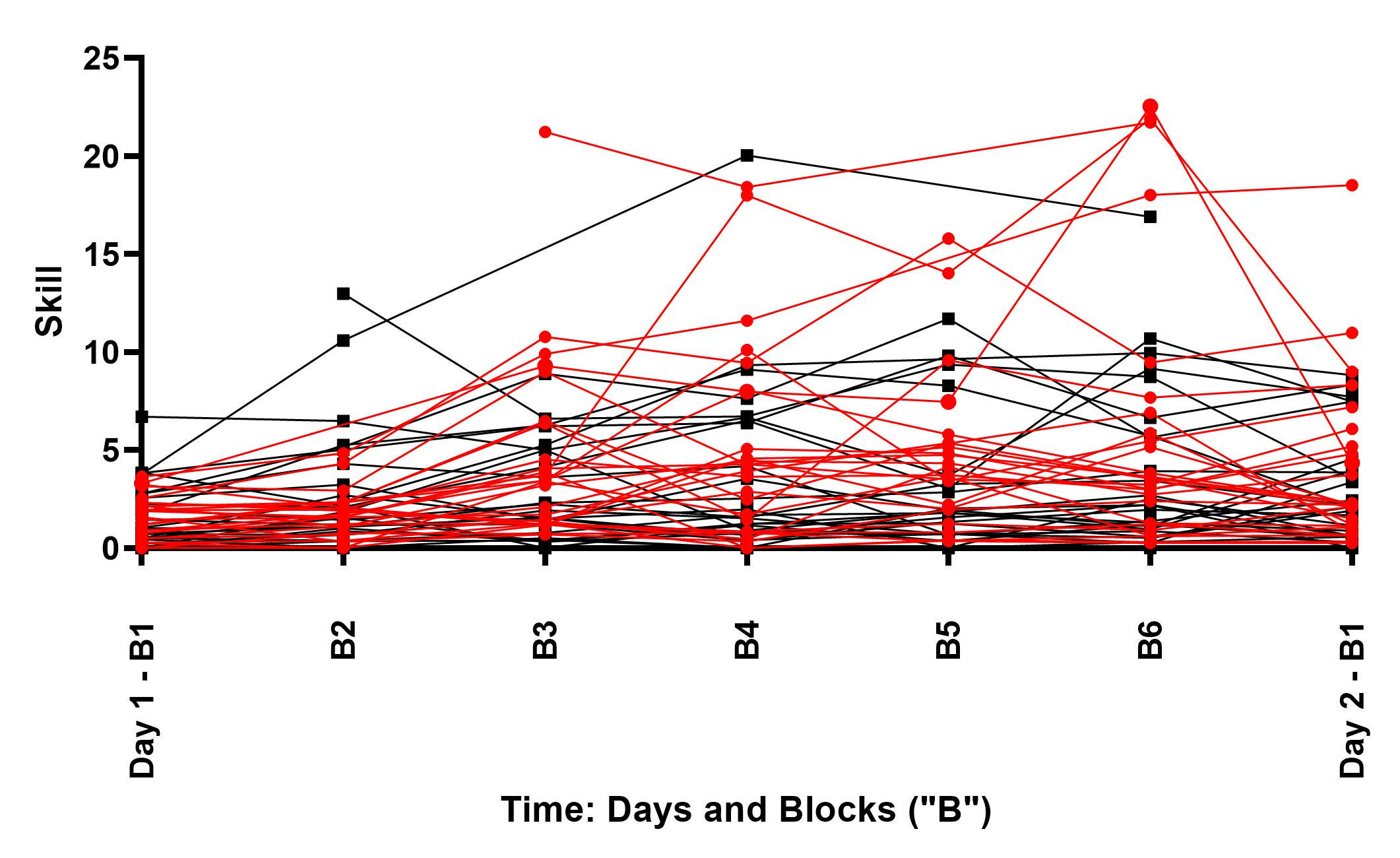

Supplement: S1 Fig — Mean skill performance (y-axis) for each block of practice across days (x-axis). Each line represents one participant’s mean skill performance. Red represent data for the anodal transcranial direct current stimulation group; black represent data for the sham transcranial direct current stimulation group. (TIF) [file pone.0269851.s001.tif]
