# Supplementary material for: Transcranial direct current stimulation leads to faster acquisition of motor skills, but effects are not maintained at retention
Source: PLoS One. 2022 Sep 13;17(9):e0269851. doi: 10.1371/journal.pone.0269851 (PMC9469971; doi:10.1371/journal.pone.0269851)
Supplement: S1 Table — Mild adverse events reported at debriefing after the session on Day 1 for both anodal and sham transcranial direct current stimulation (tDCS) groups. Participants were also asked if they thought their performance was affected by the tDCS. Participants scored according to the following scale: 1 = none; 2 = mild; 3 = moderate; 4 = considerable; 5 = strong. Data represent mean (standard deviation). (DOCX) [file pone.0269851.s002.docx]

**S1 Table. Reported mild adverse events of transcranial direct current stimulation**

| **Side effect** | **Anodal tDCS Group** | **Sham tDCS Group** |
| --- | --- | --- |
| Itching | 2.50 (1.20) | 1.76 (0.90) |
| Tingling | 2.38 (0.80) | 2.15 (0.88) |
| Pinching | 1.35 (0.69) | 1.27 (0.83) |
| Pain | 1.19 (0.40) | 1.38 (0.80) |
| Burning | 1.81 (0.85) | 2.12 (1.24) |
| Warmth | 1.81 (0.85) | 1.92 (0.89) |
| Fatigue | 1.5 (0.95) | 1.38 (0.70) |
| Headache | 1.15 (0.46) | 1.11 (0.33) |
| Dizziness | 1.12 (0.43) | 1.00 (0.00) |
| Discomfort | 1.69 (0.83) | 1.62 (0.57) |
| Performance affected | 1.31 (0.55) | 1.38 (0.57) |

Side effects reported at debriefing after the session on Day 1 for both anodal and sham transcranial direct current stimulation (tDCS) groups. Participants were also asked if they thought their performance was affected by the tDCS. Participants scored according to the following scale: 1=none; 2=mild; 3=moderate; 4=considerable; 5=strong. Data represent mean (standard deviation).
