# Supplementary material for: Transcranial direct current stimulation leads to faster acquisition of motor skills, but effects are not maintained at retention
Source: PLoS One. 2022 Sep 13;17(9):e0269851. doi: 10.1371/journal.pone.0269851 (PMC9469971; doi:10.1371/journal.pone.0269851)
Supplement: S2 Table — Mean (SD) ratings for factors that could affect performance, measured before (Pre) and after (Post) the session on Day 1 for both anodal and sham transcranial direct current stimulation (tDCS) groups. Sleep quality was rated on the following scale: 1 = poor; 2 = adequate; 3 = normal; 4 = good; 5 = excellent. The visual analogue scale (VAS) on a range from 1 to 7 was used to assess other factors. Alertness was rated as 1 = low alertness to 7 = high alertness; pain was rated as 1 = no pain to 7 = severe pain; fatigue was rated as 1 = not tired to 7 = extremely tired; muscle soreness was rated as 1 = no muscle soreness to 7 = muscles extremely sore. (DOCX) [file pone.0269851.s003.docx]

**S2 Table. Factors that may affect performance**

|  | **Anodal tDCS Group** | **Sham tDCS Group** |
| --- | --- | --- |
| Hours of sleep for previous night | 6.79 (1.47) | 6.76 (1.32) |
| Sleep Quality | 3.15 (1.01) | 2.88 (1.14) |
| Pre-VAS-Alertness | 5.42 (1.17) | 5.27 (0.92) |
| Post-VAS-Alertness | 5.08 (1.57) | 4.73 (1.28) |
| Pre-VAS-Pain | 1.65 (0.98) | 1.35 (0.98) |
| Post-VAS-Pain | 1.65 (0.80) | 1.42 (1.06) |
| Pre-VAS-Fatigue | 2.81 (1.33) | 2.69 (1.23) |
| Post-VAS-Fatigue | 3.04 (1.11) | 3.08 (1.46) |
| Pre-VAS-Muscle Soreness | 2.31 (1.85) | 1.62 (0.94) |
| Post-VAS-Muscle Soreness | 2.96 (1.71) | 2.38 (1.36) |

Mean (SD) ratings for factors that could affect performance, measured before (Pre) and after (Post) the session on Day 1 for both anodal and sham transcranial direct current stimulation (tDCS) groups. Sleep quality was rated on the following scale: 1=poor; 2=adequate; 3=normal; 4=good; 5=excellent. The visual analogue scale (VAS) on a range from 1 to 7 was used to assess other factors. Alertness was rated as 1=low alertness to 7=high alertness; pain was rated as 1=no pain to 7=severe pain; fatigue was rated as 1=not tired to 7=extremely tired; muscle soreness was rated as 1=no muscle soreness to 7=muscles extremely sore.
