# Supplementary material for: Transcranial direct current stimulation leads to faster acquisition of motor skills, but effects are not maintained at retention
Source: PLoS One. 2022 Sep 13;17(9):e0269851. doi: 10.1371/journal.pone.0269851 (PMC9469971; doi:10.1371/journal.pone.0269851)
Supplement: S3 Table — The Positive and Negative Affect Schedule (PANAS) to screen for mood was administered before (Pre) and after (Post) the session on Day 1 for both anodal and sham transcranial direct current stimulation (tDCS) groups. Data represent mean (SD). A higher “positive” score indicates a more positive affect; a lower “negative” score indicates less of a negative affect. Scores range from 10–50. (DOCX) [file pone.0269851.s004.docx]

**S3 Table. Self-reported measures of affect**

| **Positive and Negative**  **Affect Schedule** | **Anodal tDCS Group** | **Sham tDCS Group** |
| --- | --- | --- |
| Pre-positive | 30.23 (8.61) | 31.38 (7.08) |
| Post-positive | 27.62 (9.68) | 29.19 (8.39) |
| Pre-negative | 12.46 (3.20) | 12.46 (3.30) |
| Post-negative | 12.08 (3.57) | 11.04 (1.59) |

The Positive and Negative Affect Schedule (PANAS) to screen for mood was administered before (Pre) and after (Post) the session on Day 1 for both anodal and sham transcranial direct current stimulation (tDCS) groups. Data represent mean (SD). A higher “positive” score indicates a more positive affect; a lower “negative” score indicates less of a negative affect. Scores range from 10-50.
